# Supplementary material for: Caring-Healing Modalities for Emotional Distress and Resilience in Persons with Cancer: A Scoping Review
Source: Nurs Rep. 2025 Sep 10;15(9):334. doi: 10.3390/nursrep15090334 (PMC12472820; doi:10.3390/nursrep15090334)
Supplement: Supplementary file 1 [file nursrep-15-00334-s001.zip › nursrep-3823664-supplementary.pdf]

nursing reports

MDPI

Table S1. Search strategy for each database.

**PubMed**	(("cancer s"[All Fields] OR "cancerated"[All Fields] OR "canceration"[All Fields] OR "cancerization"[All Fields] OR "cancerized"[All Fields] OR "cancerous"[All Fields] OR "neoplasms"[MeSH Terms] OR "neoplasms"[All Fields] OR "cancer"[All Fields] OR "cancers"[All Fields] OR ("neoplasm s"[All Fields] OR "neoplasms"[MeSH Terms] OR "neoplasms"[All Fields] OR "neoplasm"[All Fields]) OR "oncolog*"[All Fields]) AND ("emotional distress"[All Fields] OR "psychological distress"[All Fields] OR ("anxiety"[MeSH Terms] OR "anxiety"[All Fields] OR "anxieties"[All Fields] OR "anxiety s"[All Fields]) OR ("depressed"[All Fields] OR "depression"[MeSH Terms] OR "depression"[All Fields] OR "depressions"[All Fields] OR "depression s"[All Fields] OR "depressive disorder"[MeSH Terms] OR ("depressive"[All Fields] AND "disorder"[All Fields]) OR "depressive disorder"[All Fields] OR "depressivity"[All Fields] OR "depressive"[All Fields] OR "depressively"[All Fields] OR "depressiveness"[All Fields] OR "depressives"[All Fields]) OR "mental health"[All Fields]) AND ("resilience, psychological"[MeSH Terms] OR ("resilience"[All Fields] AND "psychological"[All Fields]) OR "psychological resilience"[All Fields] OR "resilience"[All Fields] OR "resiliences"[All Fields] OR "resiliencies"[All Fields] OR "resiliency"[All Fields] OR "resilient"[All Fields] OR "resilients"[All Fields])) AND (clinicaltrial[Filter] OR randomizedcontrolledtrial[Filter])
**CINAHL Full Text**	(((cancer OR neoplasm OR oncolog*)) AND (("emotional distress" OR "psychological distress" OR anxiety OR depression OR "mental health"))) AND ((resilience OR "psychological resilience"))<br><br>Filters applied: Randomized Controlled Trial, All Adults
**PsychINFO**	Any Field: cancer OR Any Field: neoplasm OR Any Field: oncolog* AND Any Field: "emotional distress" OR Any Field: "psychological distress" OR Any Field: anxiety OR Any Field: depression OR Any Field: "mental health" AND Any Field: resilience OR Any Field: "psychological resilience" AND Age Group: Adulthood (18 yrs & older) AND Methodology: Clinical Trial
**Web of Science**	cancer OR neoplasm OR oncology* (All Fields) AND "emotional distress" OR "psychological distress" OR anxiety OR depression OR "mental health" (All Fields) AND resilience OR "psychological resilience" (All Fields) and Clinical Trial (Search within all fields) and Randomized Controlled Trial (Search within all fields) and Controlled Clinical Trial (Search within all fields)
**Embase**	(cancer:kw OR neoplasm:kw OR oncolog*:kw) AND ('emotional distress':kw OR 'psychological distress':kw OR anxiety:kw OR depression:kw OR 'mental health':kw) AND (resilience:kw OR 'psychological resilience':kw) AND ([controlled clinical trial]/lim OR [randomized controlled trial]/lim)<br><br>Filters applied: English, ([adult]/lim OR [aged]/lim) AND 'article'/it
**Scopus**	(KEY (cancer OR neoplasm OR oncolog*) AND KEY ("emotional distress" OR "psychological distress" OR anxiety OR depression OR "mental health") AND KEY (resilience OR "psychological resilience")) AND (LIMIT-TO (EXACTKEYWORD , "Adult")) AND (LIMIT-TO (DOCTYPE , "ar")) AND (LIMIT-TO (LANGUAGE , "English")) AND (LIMIT-TO (SRCTYPE , "j"))
**LILACS**	(cancer OR neoplasm OR oncology*) AND ("emotional distress" OR "psychological distress" OR anxiety OR depression OR "mental health") AND (resilience OR "psychological resilience")<br><br>Filters applied: English, Controlled Clinical Trial

**SciELO**	(cancer OR neoplasm OR oncology*) AND ("emotional distress" OR "psychological distress" OR anxiety OR depression OR "mental health") AND (resilience OR "psychological resilience")
**CUIDEN**	("cancer")OR(("neoplasm")OR(("oncolog*")AND(("emotional distress")OR(("psychological distress")OR(("anxiety")OR(("depression")OR(("mental health")AND(("resilience")OR("psychological resilience"))))))))
**ProQuest Dissertations and Theses Global**	abstract(cancer OR neoplasm OR oncolog*) AND abstract("emotional distress" OR "psychological distress" OR anxiety OR depression OR "mental health") AND abstract(resilience OR "psychological resilience")
**DART-Europe**	cancer OR neoplasm OR oncolog* AND "emotional distress" OR "psychological distress" OR anxiety OR depression OR "mental health" AND resilience OR "psychological resilience"

Table S2. Characteristics of included sources of evidence.

References	Authors/Year of Publication/Country	Purpose	Study Design	Sample Size and Characteristics	Caring-Healing Modalities	Theoretical Framework and Usage	Outcomes and Measurements
[34]	Ye et al., 2016<br><br>China	Evaluate the efficacy of the BRBC program in improving resilience, transcendence, hope, social support, and quality of life, and reducing emotional and physical distress in female breast cancer survivors.	RCT	IG: 93 (44.1% > 50 yo); loss of follow-up: 8<br><br>CG: 82 (45.1% between 30 and 50 yo); loss of follow-up: 21<br><br>NG: 76 (42.1% between 30 and 50 yo); loss of follow-up: 36<br><br>100% female<br><br>Breast cancer	IG: BRBC program<br><br>CG: routine care, eight weekly telephone follow-ups; BRBC program after the study completion	Resilience Model for Breast Cancer<br><br>Selection of study variables	• Anxiety and depression, HADS<br>• Illness uncertainty, IUSP<br>• Pain, nausea, and fatigue (physical distress), EORTC QLQ-C30<br>• Social support, Social Support Scale<br>• Hope, HS<br>• Resilience, CD-RISC-10<br>• Transcendence, CSTS
[37]	Campo et al., 2017<br><br>USA	Examine changes in psychosocial outcomes following an MSC videoconference.	Single-arm feasibility study	N= 25 (26.9 +/- 2.12 yo); 23 completed the study<br><br>100% female<br><br>Hodgkin's lymphoma (35%),	MSC	Not explicitly stated	• Anxiety, PROMIS Anxiety v1.0 short-form<br>• Depression, PROMIS Depression v1.0 short-form

				thyroid cancer (14%), non-Hodgkin's lymphoma (12%), sarcoma (12%), ovarian cancer (9%), breast cancer (6%), leukemia (3%), CNS (3%), lung (3%) and brain cancers (3%)			• Social Isolation, PROMIS Social Isolation v2.0 short-form<br>• Body Image, BIS<br>• Resilience, BRS<br>• Posttraumatic Growth, PTGI<br>• Self-compassion, SCS<br>• Mindfulness, MAAS
[35]	Ye et al., 2017<br><br>China	Assess the effect of a mentor-based and supportive-expressive program on survival in metastatic breast cancer.	RCT	IG: 95 (49.5% between 40 and 60 yo); loss of follow-up: 13<br><br>CG: 85 (41.2% between 40 and 60 yo); loss of follow-up: 19<br><br>100% female<br><br>Breast cancer	IG: BRBC program<br><br>CG: relaxation therapy and monthly telephone follow up	Not explicitly stated	• Anxiety and Depression, HADS<br>• Resilience, CD-RISC-10<br>• Quality of Life, EORTC QLQ-C30<br>• Survival, 3- and 5-year cancer-specific survival<br>• ALI, composite score of 14 indicators: BMI, WHR, RP, SDRR, heart-beat to heart-beat, resting SBP and DBP, WBC, RBC, hemoglobin, serotonin, HC, CRP, IL-6 and CD4+/CD8+
[42]	Swainston et al., 2018<br><br>UK	Assess how improving cognitive function could reduce emotional vulnerability	Quasi experimental	IG: 32 (50+/- 6.34) yo<br><br>CG: 28 (48 +/- 5.82) yo<br><br>100% female	IG: Course of adaptive dual n-back cognitive training<br><br>CG: Non-adaptive dual 1-back task	ORBIT Model and Attentional Control Theory<br><br>CHM development and	• Anxiety, composite score derived from the anxious and distress related subscales (Anxious Arousal,

		in female survivors of breast cancer.		Breast cancer		hypothesis formulation, respectively	General Distress) of the Mood and Anxiety Scale Questionnaire and the anxious subscale (Hyperarousal) of the Cancer IOE<br>• Depression, anhedonic depression subscale of the Mood and Anxiety Scale Questionnaire<br>• Resilience, CD-RISC<br>• Rumination, RRS<br>• Worry, PSWQ
[41]	Wu et al., 2018<br><br>Taiwan	Assess the effects of a PEI on anxiety, depression, disease-specific care knowledge, self-efficacy, resilience and quality of life in patients with breast cancer during and after chemotherapy.	RCT	IG: 20 (51.2 +/- 9.18)<br><br>CG: 20 (51.2 +/- 10.71)<br><br>100% female<br><br>Breast cancer	IG: PEI<br><br>CG: traditional pamphlet education approach (consultation from nurses and information sheets)	Stress and Coping Model<br><br>CHM development and selection of study variables	• Anxiety and Depression, HADS<br>• Resilience, RS<br>• Knowledge, Disease-specific care knowledge scale<br>• Self-efficacy, Self-efficacy scale<br>• Quality of Life, EORTC QLQ-C30 and EORTC QLQ-BR23
[36]	Zhou et al., 2019<br><br>China	Examine whether a CAT delivered via a mobile device can improve psychological resilience,	RCT	IG: 66 (44.62 +/- 7.89)<br><br>CG: 66 (44.37 +/- 7.32)<br><br>100% female	IG: CAT and routine nursing care<br><br>CG: routine nursing care. (health instructions, vital signs and post-surgery complications	Roy Adaptation Model<br><br>CHM development	• Anxiety, SAS<br>• Depression, SDS<br>• Resilience, CD-RISC

		and reduce depression and anxiety symptoms.		Breast cancer	monitoring, post-surgery and drainage tube care)		
[31]	Lin et al., 2020<br><br>China	Explore the effects of AIT on improving psychological resilience, CRF, and negative emotions in patients after colon cancer surgery.	RCT	IG = 100 (78% between 50-70 yo; 36% female)<br><br>CG = 100 (72% between 50-70 yo; 32% female)<br><br>Colon cancer	IG: AIT<br><br>CG: routine care (i.e., dietary advice, infection prevention, education post-surgery, monitoring of bone marrow suppression)	Not explicitly stated	• Anxiety, SAS<br>• Depression, SDS<br>• Resilience, CD-RISC<br>• Fatigue, PFS-R
[39]	Mondanaro et al., 2021<br><br>USA	Investigate the effects of clinical music therapy on resiliency in adults undergoing infusion.	RCT	IG: 43 (55 +/- 13.05; 77% female)<br><br>CG: 44 (53.41 +/- 11.94; 80% female)<br><br>Lung, breast, or gastrointestinal cancer	Instrumental or Vocal improvisation MT	Polyvagal Theory<br><br>Explanation of the CHM mechanism of action	• Anxiety and Depression, HADS<br>• Resilience, RS<br>• Pain, VAS and CAS
[45]	Zeppegno, et al., 2021<br><br>Italy	Assess the effects of PMI on anxiety, depression, redox status, and inflammation in breast cancer patients undergoing radiotherapy.	RCT	IG: 26 (63 +/- 10.7)<br><br>CG: 29 (66.6 +/- 10.9)<br><br>Participants were between 42-85 yo.<br><br>Sex distribution not mentioned<br><br>Breast cancer	IG: PMI<br><br>CG: usual care (ordinary radiation treatment for breast cancer)	Not explicitly stated	• Anxiety, STAI 1 and 2<br>• Depression, MADRS, BDI-II<br>• Resilience, RSA<br>• Quality of life, SF-36<br>• GSH, MDA (measured as TBARS), IL-6, TNFα, retinol, α- and γ-tocopherol, lycopene, β-carotene, CRP
[43]	Sakki et al., 2022	Examine the effect of a	Pilot, quasi-	N = 23 (55 yo)	MBSR program	Not explicitly stated	• Symptoms of anxiety, BAI

	Finland	mindfulness program on mental well-being, psychological symptoms, and biomarkers among breast cancer survivors with high depressive symptoms.	experimental	Sex distribution not mentioned<br><br>Breast cancer			• Symptoms of depression, BDI-II<br>• Resilience, RS<br>• Self-compassion, SCS-SF<br>• Mindfulness skills, FFMQ<br>• Psychological stress, PSS-10<br>• Quality of life, WHOQOL-BREF–questionnaire<br>• Insomnia, ISI<br>• Stress biomarkers: cortisol, adrenocorticotropin, high-sensitivity CRP from blood; 24-hour cortisol from urine.
[44]	Savaş et al., 2022<br><br>Germany	Evaluate the effect of an interdisciplinary integrative oncology group-based program on resilience and quality of life	Prospective longitudinal study	N = 60 (86.7% between 46 and 70 yo; 95% female)<br><br>Breast (71.7%), gynecologic (15%), prostate (5%), lymphoma (5%), pancreatic (1.7%), brain (1.7%)	Interdisciplinary integrative oncology group-based program	Not explicitly stated	• Anxiety and Depression, HADS<br>• Resilience, RS-13<br>• Quality of life, EORTC-QLQ C30<br>• Distress, Distress Thermometer
[38]	Finkelstein-Fox et al., 2023<br><br>USA	Examine cross-sectional and post-treatment differences in resiliency and distress between “curvivors”	Retrospective	N = 188 (56.1 +/- 11.8 yo; 86.7% female)<br><br>Breast cancer (60.4%), hematologic (6.8%), and gynecological (6.3%)	SMART-3RP	Models of stress and coping<br><br>CHM development	• Anxiety, GAD-7<br>• Depression, PHQ-8<br>• Resilience, CES<br>• Worry, PSWQ

		and “meta-vivors” initiating a SMART-3RP.					
[32]	Liu et al., 2023<br><br>China	Explore the effects of CALM on resilience and a spectrum of psychological outcomes.	RCT	IG: 62 (49.26 +/- 10.12 yo)<br><br>CG: 62 (50.44 +/- 8.59 yo)<br><br>NCG: 48 (44.81+/- 8.04 yo)<br><br>Sex distribution not mentioned<br><br>Breast cancer	CALM	Explanation of the CHM’s mechanism of action	• Anxiety and Depression, HADS<br>• Resilience, CD-RISC<br>• Quality of life, FACT-B<br>• Perceived stress, PSS
[33]	Liu et al., 2024<br><br>China	Investigate the effectiveness of combining psychological nursing with extended nursing in patients with colorectal cancer who have undergone enterostomy.	Retrospective	OG: 39 (60.88 +/- 9.93; 14 female)<br><br>CG: 38 (61.95 +/- 9.67; 13 female)<br><br>Colorectal cancer	OG: Psychological and extended nursing.<br><br>CG: Standard nursing care	Not explicitly stated	• Anxiety, HAMA<br>• Depression, HAMD<br>• Sleep quality, PSQI<br>• Resilience, CD-RISC<br>• Self-care ability, ESCA<br>• Complications, Incidence of complications within 3 months after discharge
[40]	Nakatani et al., 2024<br><br>USA	Examine the feasibility of an expressive writing modality for hospitalized patients with AML receiving induction	Pilot RCT	IG: 3 (48.0 +/- 14.2 yo; 0% female)<br><br>CG: 5 (57.6 +/- 8.5 yo; 40% female)<br><br>AML	IG: Expressive writing<br><br>CG: neutral writing prompts, describing food, exercise, medications, and daily activities	Not explicitly stated	• Anxiety, GAD-7<br>• Depression, PHQ-8<br>• Resilience, CD-RISC-10<br>• Rumination, RRS

		chemotherapy.					• Quality of life, FACT-G questionnaire<br>• Post-writing reflection, 4-item reflection survey that measured mood, intensity of expressions, value, and meaning
[46]	Tuckey et al., 2025<br><br>Australia	Assess the feasibility of an online mental health and well-being modality for women diagnosed with breast cancer.	Pilot mixed-methods uncontrolled study	19 (45.7 +/- 7.74 yo)<br><br>100% female<br><br>Breast cancer	BWP	Not explicitly stated	• Anxiety, GAD-7<br>• Depression, PHQ-9<br>• Resilience, BRS<br>• Self-compassion, SCS-SF<br>• Mental well-being, WEMWBS<br>• Quality of life, EORCQLQ-C30.

Note: BRBC, Be Resilient to Breast Cancer; RCT, randomized controlled trial; IG, intervention group; CG, control group; NG, norm group; yo, years old; HADS, Hospital Anxiety and Depression Scale; IUSP, Illness Uncertainty Scale for Patients; EORTC QLQ-C30, European Organization for Research and Treatment of Cancer Quality of Life Questionnaire – Core 30; HS, the 8-item Hope Scale; CD-RISC-10, 10-item Connor-Davidson Resilience Scale; CSTS, Chinese version of Self-Transcendence Scale; MSC, Mindful Self-Compassion; BIS, Body Image Scale; BRS, Brief Resilience Scale; PTGI, Posttraumatic Growth Inventory; SCS, Self-Compassion Scale; MAAS, Mindful Attention Awareness Scale; ALI, Allostatic Load Index; BMI, body mass index; WHR, waist-hip ratio; RP, resting pulse; SDRR, standard deviation of R-R intervals; SBP, systolic blood pressure; DBP, diastolic blood pressure; WBC, white blood cell count; RBC, red blood cell count; HS, hormone cortisol; CRP, C-reactive protein; IL-6, interleukin-6; CD4+/CD8+, cluster of differentiation 4/cluster of differentiation 8; IOE, Impact of Events Scale; CD-RISC, Connor Davidson Resilience Scale; RRS, Ruminative Response Scale; PSWQ, Penn State Worry Questionnaire; PEI, psychoeducational intervention; RS, resilience scale; EORTC QLQ-BR23, European Organization for Research and Treatment of Cancer Quality of Life Questionnaire – Breast Cancer Module; CAT, cyclic adjustment training; SAS, Self-Rating Anxiety Scale; SDS, Self-Rating Depression Scale; AIT, attention and interpretation therapy; CRF, cancer-related fatigue; PFS-R, Revised Piper Fatigue Scale; MT, music intervention; RS, resilience scale; VAS, visual analog scale; CAS, body pain scale; PMI, psychotherapy with music intervention; STAI, State-Trait Anxiety Inventory; MADRS, Montgomery–Asberg Depression Rating Scale; BDI-II, Beck Depression Inventory II; RSA, Resilience Scale for Adults; SF-36, Short Form-36; GSH, Glutathione; MDA, malondialdehyde; IL-6, interleukin 6; TNF α, tumor necrosis factor α; MBSR, Mindfulness-Based Stress Reduction; BAI, Beck Anxiety Inventory; SCS-SF, Self-Compassion Scale Short Form; FFMQ, Five Facet Mindfulness Questionnaire; PSS-10, Perceived Stress Scale-10 item; WHOQOL-BREF, World Health Organization Quality of Life Questionnaire-BREF; ISI, Insomnia Severity Index; SMART-3RP, Stress Management and Resiliency Training: Relaxation Response Resiliency Program; GAD-7, Generalized Anxiety Disorder 7-item scale; PHQ-8, Patient Health Questionnaire 8-item; CES, Current Experiences Scale; NCG, Normal control group; Managing Cancer and Living Meaningfully; FACT-B, Functional Assessment of Cancer Therapy-Breast; PSS, Perceived Stress Scale; OG, observation group; HAMA, Hamilton Anxiety Rating Scale; HAMD, Hamilton Depression Rating Scale; PSQI, Pittsburgh Sleep Quality Index; ESCA, Studies of Self-Care Ability

Scale; AML, acute myeloid leukemia; RRS, Rumination Response Scale; FACT-G, Functional Assessment of Cancer Therapy-General; BWP, Be Well Plan; PHQ-9, the 9-item Patient Health Questionnaire; BRS, Brief Resilience Scale; SCS-SF, Self-Compassion Scale - Short Form; WEMWBS, Warwick-Edinburgh Mental Well-Being Scale.

Table S3. Characteristics of caring-healing modalities included in the sources of evidence analyzed in this scoping review.

References	Authors/ Year of Publication	Caring-Healing Modality Setting	Active ingredients	Non-specific Elements	Medium	Format	Structure/Approach	Dose	Duration and Frequency
[34]	Ye et al., 2016	Outpatient setting	Education related to breast cancer (e.g. breast reconstruction, posttreatment issues, sexuality, anxiety, depression, uncertainty, diet and nutrition, music therapy, emotion management, positive mind therapy, traditional Chinese medicine, Taichi).	Not identified	Group-based and one-on-one; person-dependent<br><br>Verbal/written	In-person, phone calls<br><br>Techniques included didactic lectures and group discussions on the following themes: surgery and treatment, physical therapy, emotional distress, nausea, diet and nutrition, traditional Chinese Medicine, Taichi practice, music and relaxation techniques, sexuality, restoration, to be better, and renewal.	Standardized<br><br>Curriculum with tailored mentor-mentee matching	3 hours per session	12 months; 8 weekly sessions in first 2 months, plus 3 follow-ups at 2, 6, and 12 months

			Peer support<br><br>Group discussions						
[37]	Campo et al., 2017	Participants' homes or private location	MSC, self-esteem, gratitude, and self-appreciation	Quiet environment (headphones), control of distractions, protection of privacy, interaction among members on Facebook secret groups, assistance with technical issues while using online platform, reminders	Group-based, person dependent<br><br>Verbal/written	Online/remote (videoconference), including several techniques: didactic instruction, experiential activities (e.g., compassionate friend meditation, body scan, here-and-now stone, affectionate breathing meditation, loving kindness meditations, soften-soothe allow meditation, gratitude phone photos), introduction of different meditations and daily tools, group discussion, and interaction on Facebook	Standardized	90-minute sessions	Once per week for 8 weeks
[35]	Ye et al., 2017	Outpatient setting	Education related to breast cancer (e.g., surgery and	Not identified	Group-based<br><br>Verbal/written	In-person<br><br>Techniques included didactic lectures and group discussions	Standardized	120 minutes per session	12 months; weekly sessions

			treatment, physical therapy, emotional distress, nausea, diet and nutrition, traditional Chinese Medicine, Taichi practice, music and relaxation techniques, sexuality, restoration, to be better, and renewal)<br><br>Peer support						
[42]	Swainston et al., 2018	Home	Cognitive training	Not identified	Individual-based<br><br>Verbal/written	Online<br><br>Techniques included adaptive dual n-back cognitive training tasks	Standardized	30 minutes per session	12 days across 2-week period; daily sessions
[41]	Wu et al, 2018	Cancer Medical Center	PEI consisting of an educational manual addressing	Supervision by healthcare professionals, use of a self-	Individual-based<br><br>Verbal/written	In person; techniques included interaction between healthcare professionals and patients (telephone	Standardized CHM plan based on educational and support components	1 hour per session	6 sessions during five chemotherapy treatments

			depression, anxiety, disease-specific care knowledged ge, self-efficacy, and resilience, and a self-assessment of learning	directed videotape, educational information and materials		consultations), self-directed videotape, educational manual			
[36]	Zhou et al., 2019	Hospital/home	Deep breath training, music listening, anti-cancer stories (reading/listening/watching), self-reflection, shared decision-making	Routine nursing care, health instruction, vital signs and post-surgery complications monitoring, post-surgery and drainage tube care	Group-based, person-dependent<br><br>Verbal/written	Online (via WeChat), in person (nurse-to-patient instruction)	Standardized	30-60 minutes per session for nurse-to-patient instruction before surgery; 20 minutes per session for relaxed deep breath training before surgery; 10 minutes per session for relaxed deep breath training three times per	From hospital admission to 12 weeks follow-up; relaxed deep breath training three times per day and once before surgery; music listening and anti-cancer stories (reading/listening/watching) three times per day; re-introspect once per week

								day after surgery; 20-30 minutes per session for music listening and anti-cancer stories (reading/listening/watching) three times per day after surgery.	
[31]	Lin et al., 2020	Hospital and home	Transcendental meditation, emotional control, cultivation of appreciation, mindfulness, acceptance, support from peers, and commitment therapy	Nurse supervision and follow-up, personal file establishment, incentives for continuous practice (text message), maintaining peer connection, communication with interventionists	Group based person depended<br><br>Verbal/written	Face-to-face<br><br>Techniques included didactic instruction, group meetings, emotional diaries, WeChat emotion management applet, video sharing, patient workshops	Tailored according to the special needs of the patient	30 minutes per session	Daily for 10 weeks
[39]	Mondanaro et al., 2021	Hospital	Instrumental or vocal music	Not identified	Individual-based	In-person<br><br>Techniques included warm up,	Standardized	20 minutes per session	3 sessions over 1 to 3 months

					Verbal, auditory	improvisation (melody, harmony, timbre, and rhythmic idioms), discussion of therapeutic goals and themes or issues identified in the music or self-disclosure			
[45]	Zeppegno , et al., 2021	Quiet room at a hospital	Psychodynamic psychotherapy, MT, peer support	Supportive role of residents, quietness, privacy	Group-based<br><br>Verbal/written	In-person<br><br>Techniques included music listening, song lyric analysis, sharing of emotions and memories, group discussions	Standardized	1 hour per session	6 weeks; weekly sessions
[43]	Sakki et al., 2022	Between-session practices done at home; group sessions not clearly stated	Mindfulness home practice, including body scan, breathing exercises, mindful movements and yoga practice, awareness	Silent retreat, diary, audio recordings describing nature and content of mindful practices	Group-based<br><br>Verbal/written	Informal home practices, group sessions, and diary of independent mindfulness practice	Standardized	2.5 hours per session, 45 minutes for home practices	8 weeks; weekly sessions, in addition to between-session practices and one day long silent retreat
[44]	Savaş et al., 2022	Outpatient setting	Neurocognitive restructuring, recommendations for diet,	Not identified	Group-based<br><br>Verbal, auditory, tactile	In-person<br><br>Music therapy, manual therapies	Standardized	5 hours per session, including a 2-hour break	10 weeks; weekly sessions

			exercise, stress management, relaxation, naturopathic self-help strategies, and psychosocial support						
[38]	Finkelstein-Fox et al., 2023	Not clearly stated	Stress-awareness, stress-coping, and stress-buffering skills	Not clearly stated	Group-based<br><br>Person-depended<br><br>Verbal/written	In-person, videoconferencing<br><br>Program manual<br><br>Mind-body techniques that elicit the RR (i.e., meditation, breath awareness), positive psychology (i.e., shifting focus to positive experiences), and cognitive behavioral therapy (i.e., re-structuring negative thoughts)	Tailored	Not clearly stated	Once per week for 8 or 9 weeks
[32]	Liu et al., 2023	Hospital	Guidance for symptom management, discussion of changes brought by the disease, psychotherapy	Trusting relationship, education regarding adverse effects of illness and treatment	Individual-based<br><br>Visual/auditory, verbal	In-person<br><br>Techniques included dialogue, didactic instruction, virtual reality therapy	Tailored to the participants' needs	At least 30 minutes per session	12 weeks; six sessions over 12 weeks

			to clarify the purpose and meaning of existence, discussion of concerns about the future and understanding of death						
[33]	Liu et al., 2024	Outpatient setting and home	Education, instillation of hope and confidence, meaning therapy, family association and peer support, emotional expression, and extended care (follow-up)	Personal file establishment, maintaining communication with interventionists, and encouragement to ask questions	Individual- and group-based, person-depended<br><br>Verbal/written	Face-to-face/in-person, telephone, and online<br><br>Techniques included didactic instruction, group meetings, emotional expression, WeChat groups, video sharing	Tailored to the participants' needs	Not clearly stated	Once per week, not clearly stated for how long
[40]	Nakatani et al., 2024	Hospital	Emotional expression<br><br>Post writing reflections	Instructions	Individual-based<br><br>Verbal (coaching)/written	In-person and remote (Zoom and phone)<br><br>Techniques included writing prompts and coaching	Standardized	1-hour sessions	4 sessions over 2 weeks

[46]	Tuckey et al., 2025	Outpatient setting and home	Psychoeducation, self-reflection, and sharing	Awareness of participant's mental health (survey); having a support person in the program	Group-based<br>Verbal/written	Online or face-to-face/in-person, mobile app	Tailored (developing own well-being plan)	2 hours per session	5 weeks; weekly sessions

Note: MSC, Mindfulness, self-compassion; PEI, Psychoeducational intervention; MT, music intervention; RR, relaxation response; CALM, Managing Cancer and Living Meaningfully.
